# Antioxidant Role of Kaempferol in Prevention of Hepatocellular Carcinoma

**DOI:** 10.3390/antiox10091419

**Published:** 2021-09-05

**Authors:** Nidhi Sharma, Subhrajit Biswas, Noura Al-Dayan, Alaa Saud Alhegaili, Maryam Sarwat

**Affiliations:** 1 Amity Institute of Pharmacy, Amity University, Noida 201313, Uttar Pradesh, India; nidhi13995@gmail.com; 2 Amity Institute of Molecular Medicine & Stem Cell Research, Amity University, Noida 201313, Uttar Pradesh, India; sbiswas2@amity.edu; 3 Medical Laboratory Department, Applied Medical Science, Prince Sattam bin Abdul Aziz University, Al-Kharj 16278, Saudi Arabia; n.aldayan@psau.edu.sa (N.A.-D.); a.alhegaili@psau.edu.sa (A.S.A.)

**Keywords:** free radicals, oxidative stress, HCC, anti-oxidants, ER stress, kaempferol

## Abstract

Reactive oxygen species (ROS) are noxious to cells because their increased level interacts with the body’s defense mechanism. These species also cause mutations and uncontrolled cell division, resulting in oxidative stress (OS). Prolonged oxidative stress is responsible for incorrect protein folding in the endoplasmic reticulum (ER), causing a stressful condition, ER stress. These cellular stresses (oxidative stress and ER stress) are well-recognized biological factors that play a prominent role in the progression of hepatocellular carcinoma (HCC). HCC is a critical global health problem and the third leading cause of cancer-related mortality. The application of anti-oxidants from herbal sources significantly reduces oxidative stress. Kaempferol (KP) is a naturally occurring, aglycone dietary flavonoid that is present in various plants (*Crocus sativus, Coccinia grandis, Euphorbia pekinensis*, varieties of *Aloe vera*, etc.) It is capable of interacting with pleiotropic proteins of the human body. Efforts are in progress to develop KP as a potential candidate to prevent HCC with no adverse effects. This review emphasizes the molecular mechanism of KP for treating HCC, targeting oxidative stress.

## 1. Introduction

**Figure 1:**
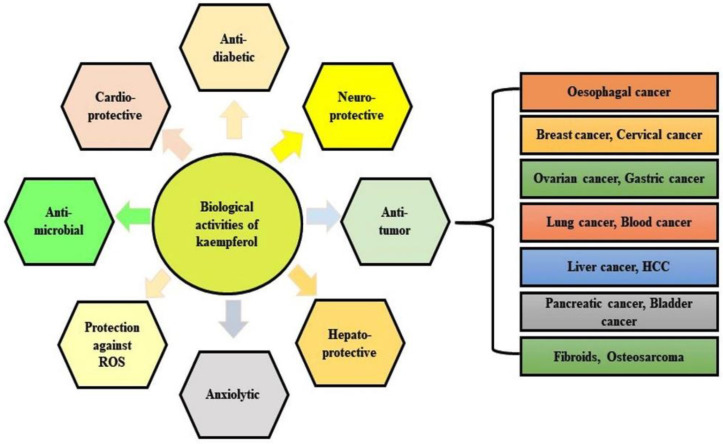
Pharmacological activities of kaempferol.

Kaempferol is a yellow-colored dietary flavonoid, present in numerous fruits and vegetables including apples (*Malus domestica*), aloe (*Aloe vera*), beans (*Phaseolus vulgaris*), broccoli (*Brassica oleracea*), carrot (*Daucus carota*), gooseberry (*Ribes uva-crispa*), strawberries (*Fragaria × ananassa*), saffron (*Crocus sativus*), tea (*Camellia sinensis*), and honey (*Apis mellifera*) [1,2,3,4,5]. It is a tetrahydroxyflavone that has hydroxy groups located at positions 3, 5, 7, and 40 [6]. Kaempferol and its glycosylated derivatives are found to possess cardioprotective [7], neuroprotective [8], anti-inflammatory [4], antidiabetic [9], antioxidant [10], antimicrobial, [11] and anti-cancer activities [12,13] (Figure 1). Kaempferol gets absorbed by the small intestine due to its lipophilicity [14] or either by passive absorption, facilitated diffusion, or active transport [15]. Kaempferol, being a polyphenolic neutraceutical compound, exhibits high cytotoxicity, and thus has a promising role in cancer therapy. It has been demonstrated to invoke several mechanisms in the regulation of cancer cells. Cancer prevention is largely accomplished by inducing apoptosis, inhibiting cell proliferation [16], and promoting cell cycle arrest mainly in the G2/M phase [17]. Researchers have reported that kaempferol reduces cell viability and proliferation in the lungs [18], and colorectal cancer [19]. It inhibits the cell viability in HCC in a dose- and time-dependent manner [20]. Epidemiological data showed that a good intake of kaempferol is connected with low incidences of liver cancer [21]. Encouragingly, a kaempferol-rich diet has reduced the risk of cancer in smokers [22]. It has proved to be a better candidate molecule in increasing the efficacy of other anticancer drugs. Kaempferol, in combination with sorafenib (at subtoxic concentration), enhances the efficacy of sorafenib chemotherapy [23]. It is a potent scavenger of ROS that works by reducing cellular oxidative stress [24]. It augments the antioxidant potential of normal cells via modulating heme oxygenase (HO)-1 expression and mitogen-activated protein kinase (MAPK) pathways [25]. The HO-1 is a redox-sensitive inducible enzyme whose overexpression enhances cell resistance to oxidative injury.

Free radicals are moieties with one or more unpaired electron in atomic or molecular orbitals formed during a variety of biochemical reactions and cellular functions [26]. ROS are the active metabolites of healthy cells, which include free radicals such as superoxide (O_2_^−^) and hydroxyl radical (OH^•^), as well as nonradical species, such as hydrogen peroxide (H_2_O_2_) [27]. The sequential reduction of oxygen leads to the genesis of these species. The physiological concentration of ROS is essential in carrying out vital cellular processes including proliferation, apoptosis, cell cycle arrest, and cell senescence, whereas high ROS flux damages cell macromolecules including proteins, lipids, and nucleic acids (DNA and RNA) [28]. Therefore, the smooth conduction of all metabolic functions requires a balanced redox state. Overproduction of ROS due to endogenous (e.g., mitochondria, peroxisomes, and oxygen-handling enzymes) and exogenous reactions (e.g., UV, heavy metals, and micronutrients) or inefficient/exhausted antioxidants leads to oxidative stress [27]. It may lead to the development of various chronic diseases such as cardiovascular diseases [29,30], neurodegenerative diseases [31,32], allergy [33], and carcinogenesis [34].

HCC is the most lethal type of cancer, and is the third leading cause of cancer-related mortality worldwide [35,36]. Risk factors such as hepatitis B virus (HBV), hepatitis C virus (HCV), aflatoxin-contaminated food, cirrhosis, diabetes mellitus, obesity, alcohol abuse, smoking, and non-alcoholic fatty liver diseases (NAFLD) are involved directly and indirectly in the pathogenesis of HCC [37,38]. Management of HCC patients depends on the stage of the tumor. Surgery is the main curative therapy for HCC, but there are very high chances of tumor recurrence in patients with HCC [39]. The treatment options comprise repeated liver resection, transarterial therapy, ablative therapy, and systemic medical therapies [40]. A recent study proposed the beneficial role of salvage liver transplant in treating HCC recurrence, but this remains controversial due to organ shortage and the overall low rate of patients that may fulfill transplant criteria at the time of recurrence [41]. Many scientists have reported the better long-term effects of redo surgery over thermoablation. Palliative treatment of HCC includes trans-arterial chemo-embolization (TACE), targeted molecular therapy, and systemic medical therapies such as Sorafenib treatment, etc. [42]. HCC is a multi-stage process that involves various complex pathways in its pathogenesis, including RAF/ERK/MAPK, PI3K/Akt/Mtor, Ras and JAK-STAT, Wnt- and RB1-dependent signaling cascade [43]. However, oxidative stress emerged as a key player in the development and progression of HCC [44]. Dysregulation of ROS-producing and ROS-scavenging enzymes contributes to the development of HCC leading to poor patient survival. This review summarizes the mechanism of the antioxidant potential of kaempferol in treating HCC.

## 2. Regulated Cell Death and Kaempferol

Regulated cell death (RCD) is beneficial in maintaining the organism’s hemostasis. Autophagy and apoptosis are the two important parts of RCD [45]. Autophagy is an evolutionary conserved cellular process targeting the damaged cells and organelles in lysosomal degradation. Autophagy is induced in response to the metabolic crisis and damaged organelles [46]. Apoptosis, or programmed cell death, governs the autonomous removal of infected and damaged cells. Activation and suppression of the two arms of RCD (apoptosis and autophagy) have been suggested as methods for curing liver cancer, including HCC [47,48,49,50]. Several hypotheses have been considered regarding the antitumor potential of kaempferol via RCD. Kaempferol is reported to induce autophagic cell death against SK-Hep1 (human hepatic cancer cell line) via AMPK and AKT signaling pathways [17]. Kaempferol inhibits cell proliferation, metastasis, invasion and induces apoptosis in HepG2 liver cancer cells by reducing the expression of miRNA [51]. Kaempferol, in combination with luteolin, induces apoptosis and causes cell cycle arrest at the G2/M phase, thus preventing cell migration and invasion [14].

## 3. Oxidative Stress (OS) in Hepatocarcinogenesis

Various mechanisms involved in hepatocarcinogenesis include the attenuation of tumor suppressor function, oncogene activation, and oxidative stress [52].

Oxidative stress could be promoted by any dangerous or inflammatory signal which damages hepatocytes, promotes pathological polyploidization, and triggers inflammation [53]. Increased levels of ROS and oxidative stress promote genetic and epigenetic alterations which contribute to the regulation of onco-suppressor, onco-promoter, and several proinflammatory genes involved in the development of HCC [54]. Additionally, they also promote the migration, invasion, and metastasis of HCC for different etiologies [55]. More the 80-90% of HCC cases are associated with chronic hepatic inflammation, non-alcoholic steatohepatitis (NASH), and hepatitis B virus (HBV), and hepatitis C virus (HCV) [56].

In the hepatocytes, ROS cause the activation of several cellular pathways including mitogen-activated protein kinase (MAPK), nuclear factor-kB (NF-kB), phosphatidylinositol 3- kinase (PI3K), p53, b-catenin/Wnt, and angiogenesis. Notably, these pathways play a vital role in mutagenesis, tumor promotion, and progression [57], and for this reason, oxidative stress has a strong connection with hepatocarcinogenesis.

### 3.1. HBV and HCV Related HCC and Oxidative Stress

Chronic HBV and HCV infection are often associated with the development of cirrhosis, and HCC [58]. These unrelated viruses belong to two different viral families and exhibit strong hepatotropism, but their molecular mechanism to produce HCC is still under investigation. Some researchers have found that the viral encoded proteins alter the cellular phenotype and host gene expression, which is the hallmark of cancer [59]. HBV- and HCV-related fibrosis and chronic inflammation of the liver are induced by OS, which eventually contributes to the development of HCC. HBV infection leads to the activation of macrophages or Kupffer cells to produce proinflammatory cytokines, including IL-1β, IL-6, and TNF-α [60]. Irregular cytokine generation and ROS production have an influential role in hepatocarcinogenesis. The HBV genome encodes a variety of gene products, including a multifunctional HBx protein, which has carcinogenic potential. This protein promotes replication of the virus and protects the virus-infected cells from damage [61]. This process takes place in hepatocytes only, which further progresses to HCC [62].

Genetic mutations found in the samples from HBV patients have a strong correlation with the initiation and development of liver cancer [63]. Recent studies proved that the mutant genes and their products accumulate in the endoplasmic reticulum (ER) and promote carcinogenesis through ER stress and ROS production. On the other hand, during HCV infection, immunomodulatory molecules such as programmed cell death protein ligand-1 (PD-L1) become activated, affecting inflammatory signaling pathways. Continuous inflammation leads to the development of HCC via PKR, STAT3, and TNFR pathways [64]. Moreover, serological markers and iron accumulation are usually elevated during chronic HCV infection, and excessive bivalent iron is strongly toxic, leading to the induction of Fenton’s reaction and ROS [65]. Thus, oxidative stress plays an important role in HBV- and HCV-related liver cancer development.

### 3.2. Non-Alcoholic Steatohepatitis (NASH) Related HCC and OS

NASH is a chronic liver injury causing steatosis, inflammation, and progressive fibrosis, ultimately leading to cirrhosis and HCC [66]. As proposed by Day and James, the pathogenesis of NASH is a two-hit theory [67]. The first hit includes the progression of steatosis, correlated with the accumulation of triglycerides in the liver cells. The second hit includes a wide variety of cellular stress factors, e.g., gut-derived stimulation, intestinal circumstances, apoptosis, oxidative stress, and ER stress, etc. [68]. Overconsumption of carbohydrates or saturated fatty acids and less polyunsaturated fatty acids leads to lipid accumulation in hepatocytes. The adipose tissues release adiponectin, resistin, and tumor necrosis factor-alpha (TNF-α), leading to inflammation of the cells, induction of mitochondrial destruction, and ROS generation [69]. Among the above-mentioned factors, adiponectin is a fat factor that modulates cell proliferation, inhibits cancer cell growth, metastasis [70], and induces apoptosis [71]. Oxidative stress is an important process regulated by the 1L-17 protein, whose receptors are widely distributed on the surface of liver cells. Patients with an elevated level of serum IL-17 have a higher risk of early recurrence of liver cancer after surgery [72]. ROS are the metabolic by-products in hepatocytes generated due to elevated mitochondrial fatty acid oxidation and inadequate mitochondrial respiratory chain activity. The ROS level tends to increase in NASH [73] and results in the disruption of hepatic fatty acid homeostasis and accumulates non-metabolized fatty acids in the cytoplasm [74]. Thus, oxidative stress is a harmful key component causing the progression of NASH to HCC. The mechanism of oxidative stress in HBV, HCV, and NASH-related HCC is summarized in Table 1.

## 4. Antioxidant Potential of Kaempferol in Preventing HCC

Kaempferol possesses a remarkable spectrum of pharmacological activities, including antidepressant, anxiolytic, anti-inflammatory, antitumor, etc. [76,77]. Researchers have indicated the antioxidant potential of kaempferol in both in vitro and in vivo models [78]. It causes the scavenging of the free radicals and other ROS molecules, as their generation transforms the normal cells into malignant ones [79]. So, inhibition of these species alters the tumor cell phenotype. Kaempferol pre-treatment of CCl_4_ challenged mice showed normalized activities of liver enzymes [80]. Further, kaempferol 3-*O*-β-d-(2,6-di-*O*-α-l-rhamnopyranosyl) galactopyronoside (KG) pretreatment showed improvement in the level of thiobarbituric acid reactive substances in the liver, indicating that KG alleviates liver injury [80], which might be due to its antioxidant properties. Kaempferol reduces liver damage in acetaminophen-treated rats by upregulating silent information regulator 1 (SIRT1). It suppresses the acetylation of all SIRT1 targets, including PARP1, p53, NF-jB, FOXO-1, and p53 that mediate antioxidant, anti-inflammatory, and anti-apoptotic effects [81]. It also showed a hepatoprotective effect in alcohol-induced liver injury in mice by suppressing the expression of key microsomal enzyme cytochrome 2E1 (Cyp2E1) and by enhancing the protective role of the antioxidative defense system [82]. Various works citing the role of kaempferol in managing severe liver injuries are summarized in Table 2.

Several signaling pathways and molecular mechanisms have been identified that play prominent roles in reducing oxidative stress. Targeting the critical pathways which include peroxisome proliferator-activated receptor (PPAR) and nuclear factor erythroid related factor 2 (Nrf2) using kaempferol has shown a positive effect in relieving oxidative stress.

### 4.1. Peroxisome Proliferator-Activated Receptor (PPAR)

PPAR belongs to the nuclear receptor superfamily [97]. Among the various PPAR receptors identified, PPARα and PPARγ play an important role in the regulation of lipids and glucose metabolism [98]. The subtypes of PPAR receptors have been shown to be involved in the pathogenesis of HCC. Due to the increased consumption of nutrients, HCC cells experience oxygen and nutrient deficiency leading to a stressful metabolic environment [99]. PPARα acts as a master regulator of liver metabolism. Therefore, PPARα-regulated processes are involved in most liver diseases. HCC is associated with the down-regulation of PPARα receptors [99]. Thus, stimulation of PPARα is expected to treat HCC. Kaempferol, a polyphenolic compound shows the protective effect by elevating the expression of the PPARα gene and/or protein [100].

### 4.2. Nuclear Factor Erythroid Related Factor 2 (Nrf2)

**Figure 2:**
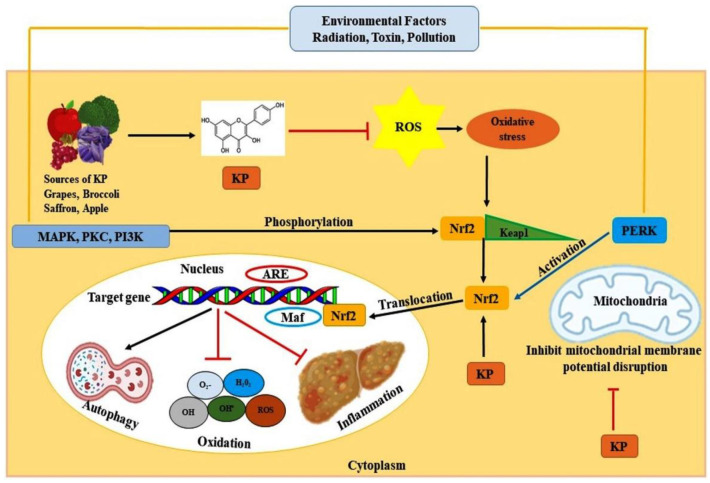
Antioxidant mechanism of KP via Nrf2-Keap1 pathway. KP inhibits the metabolism of ROS by acting on the Nrf2-Keap1 complex. The level of Nrf2 is augmented by KP after its disassociation from the complex. Nrf2 translocates to the nucleus and binds with ARE along with Maf transcription factor, which triggers the expression of the genes, inducing autophagy, inhibiting oxidation and inflammation. KP also plays a vital role in suppressing the mitochondrial membrane potential disruption and thus leading to restoration of normal physiological condition. Abbreviations: KP, kaempferol; Nrf2, nuclear factor erythroid 2 related factors 2; Keap1, Kelch-like ECH-associated protein 1.

Nrf2, a cytosolic transcription factor, is the principal regulator of cellular defense through the antioxidant machinery [101]. In normal liver cells, Nrf2 offers protective effects against oxidative stress, whereas, in the tumor cells it causes deleterious effects, encouraging the proliferation and survival of cancerous cells [102]. Under normal physiological conditions, Nrf2 and Kelch-like ECH-associated protein 1 (KEAP1) orchestrate the NRF2-dependent oxidative stress response and maintain liver homeostasis. Upon continuous stress exposure, Keap 1 is degraded in the cytoplasm. Further, Nrf2 is phosphorylated and translocated to the nucleus, forming a heterodimer with transcription factor Maf. It binds to the antioxidant response element (ARE) sequence and activates the expression of endogenous antioxidants, phase II detoxifying enzymes and transporters [103]. Nrf2 can act as the potential target for managing severe cancers including HCC [101]. Thus, kaempferol can regulate the Nrf2 transcriptional pathway and reduce cell redox homeostasis, and can play a promising role in combatting cancer [14]. Figure 2 exhibits the antioxidant role of kaempferol during HCC.

## 5. Role of Oxidative Stress in Endoplasmic Reticulum (ER) Hemostasis

**Figure 3:**
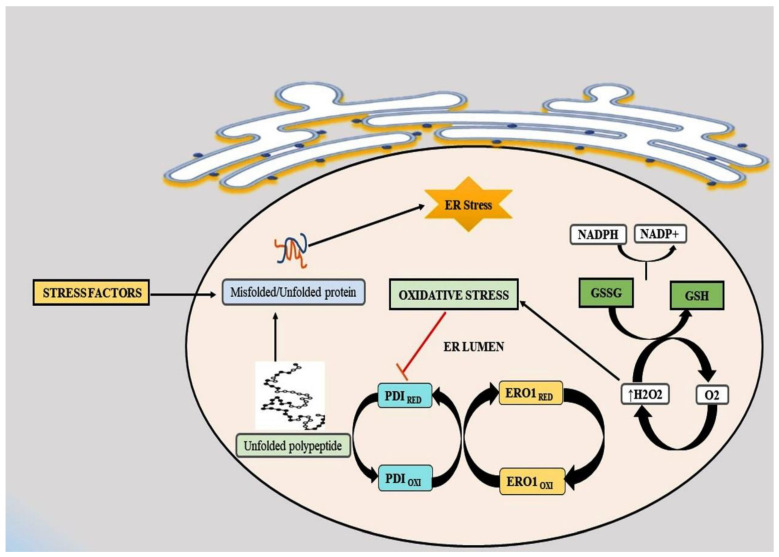
Protein folding in ER in the presence of oxidative stress. In eukaryotic cells, protein folding in ER is regulated by different proteins (PDI and ERO1). Impaired disulfide bond formation leads to the accumulation of misfolded proteins resulting in oxidative stress. Abbreviations: ER, endoplasmic reticulum; ERO1, ER oxidoreductin 1; FFA, free fatty acids; GSH, glutathione; GSSG, glutathione disulfide; NADPH, nicotinamide adenine dinucleotide phosphate; PDI, protein disulfide isomerase.

The ER is involved in protein folding, synthesis, and secretion [104,105]. Nutrient distress, pH imbalance, and, hypoxia perturb ER homeostasis, thus affecting the protein folding machinery and the generation of misfolded proteins [106]. The accumulation of misfolded proteins causes cellular damage and induces ER stress [107]. Prolonged ER stress activates a self-protective mechanism, the unfolded response (UPR). It reduces protein synthesis and enhances the expression of the ER molecular chaperones glucose-regulated protein 78 (GRP78) and GRP94 to facilitate the correct folding of proteins. UPR is the complex cellular response that is associated with the various membrane biosensors; protein kinase RNA (PKR)-like ER kinase (PERK), inositol requiring enzyme 1α (IRE1α), and activating transcription factor 6 (ATF6) [97]. Other studies have also indicated that oxidative stress is strongly connected with ER stress [108,109]. Both of them trigger various inflammatory molecules and apoptosis cascades which are involved in the pathogenesis of many diseases, specifically liver injuries. In the ER lumen, stable protein folding requires the formation of disulfide bonds between cysteine residues of the proteins [110]. Glutathione (GSH), a non-protein thiol present abundantly in eukaryotic cells, can be reduced to glutathione disulfide (GSSG), which is important in maintaining ER redox hemostasis and which ensures correct protein folding [111]. An imbalance between the glutathione (GSH/GSSH) ratio and the generation of misfolded proteins leads to the production of ROS [112]. In eukaryotes, protein folding is regulated by multifunctional chaperons and oxidoreductases (protein disulfide isomerase (PDI)). PDI accepts a pair of electrons from the cysteine residues in polypeptide substrates, resulting in its reduction and the oxidation of its substrates. Further, PDI transfers the electrons to the ER oxidoreductase 1 (ERO1), which further transfers it to the molecular oxygen and produces H_2_O_2_ to start a new cycle (Figure 3) [113].

Oxidative stress activates ER stress cascade and alleviates the expression of ER transmembrane proteins, e.g., ATF6, CHOP, and ATF4. A decrease in oxidative stress means a decrease in ER stress [114]. The correlation of oxidative stress with ER stress in liver injuries has been reported by a number of authors. Kim et al. (2018) showed that TM-induced ER stress increases MDA levels, GRP 78, and CHOP, and decreases GSH levels in liver cancer cell lines [97]. Moslehi et al. (2019) showed that TM-induced ER stress attenuates amygdalin. It can be said that amygdalin works through antioxidant machinery to combat ER stress [115]. Zhang et al. (2019) reported the pathogenic role of oxidative stress and ER stress in the early initiating stages of non-alcoholic fatty liver diseases (NAFLD) [116].

## 6. Endoplasmic Reticulum Stress Signaling Pathways

**Figure 4:**
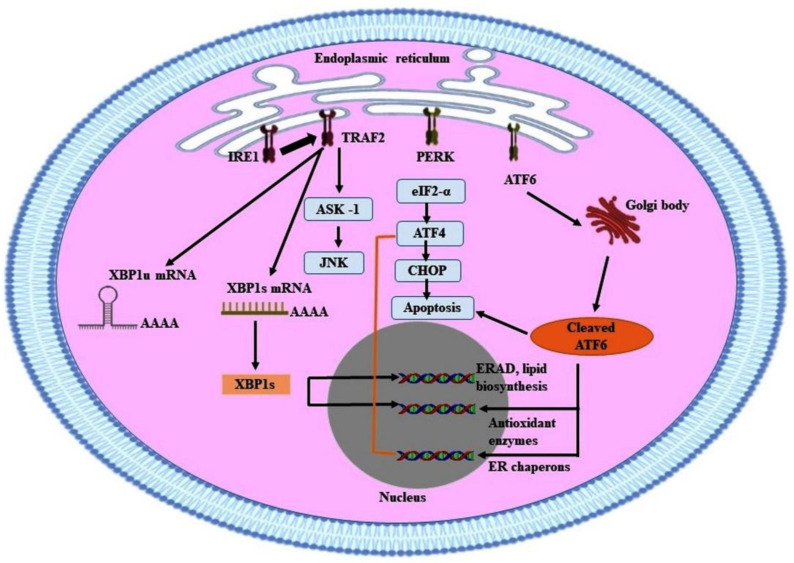
Adaptive pathways of ER stress. ER stress is induced due to the accumulation of misfolded and poorly folded proteins in the ER lumen. ER stress triggers UPR, which attenuates protein translation, enhances protein folding capacity, and thus re-establishes ER hemostasis. ER-resident chaperons interact with each other. Grp78/BiP interacts with three ER transmembrane proteins, *viz.* IRE-1α, PERK and ATF6. IRE-1α regulates the splicing of XBP1, which regulates the upregulation of ERAD. PERK phosphorylates eIF2α, which further activates ATF4 and thus increases the ER’s protein folding capacity. ATF6 gets cleaved by the specific proteases residing in the Golgi bodies, thus enhancing the expression of UPR genes and ERAD. Abbreviations: UPR, unfolded protein response; Grp78, glucose-regulated protein 78; BiP, binding protein; IRE-1α, Inositol requiring enzyme 1 α; PERK, protein kinase RNA-like ER kinase; ATF6, activation transcription factor; XBP1, X- box-binding protein; ERAD, ER-associated degradation; eIF2α, eukaryotic transcription factor 2; ATF4, Activation transcription factor.

The ER stress pathway has been considered the most efficient apoptosis signaling pathway and has a vital role in human liver cancer. IRE1α-XBP1, PERK-eIF2α-ATF4, ATF6 are the UPR signaling pathways that promote cell death in response to ER stress (Figure 4). The functions of each UPR mediator and their possible links to apoptotic signaling are discussed below.

### 6.1. IRE1α-XBP1 Pathway

IRE1 is the transmembrane type-I protein that possesses both kinase and endoribonuclease (RNAse) activities and helps in modulating ER stress [117]. IRE1 exists in two isoforms, IRE1α and IRE1β. The IRE1α is expressed extensively, whereas IRE1β expression is confined to the intestinal epithelium and gastrointestinal tract. *IRE1α*-knockout mice exhibit embryonic lethality, while *IRE1β* knockout in mice is viable. Thus, IRE1α is considered a positive regulator for mammalian cell survival [118]. During ER stress, IRE1α disassociates from GRP78/Bip [119], undergoes dimerization, and autophosphorylation. X-box binding protein 1 (XBP-1) mRNA is the first substrate described for IRE1α endonuclease activity [120], which stimulates the non-conventional splicing of XBP-1 mRNA to produce its active form that is spliced XBP-1 [121]. This activated form of XBP1 encourages the expression of ER quality-control genes, thus enhancing the protein folding capacity of the ER. Spliced XBP1 modulates the expression of genes involved in protein folding, secretion, redox homeostasis, oxidative stress response, and ER-associated degradation (ERAD) [122]. IRE1α-XBP1 signaling has been reported to possess a prominent role in human cancer including HCC [123].

### 6.2. PERK-eIF2α-ATF4 Pathway

The enzyme protein kinase R-like endoplasmic reticulum kinase (PERK) is an ER transmembrane protein that is associated with BiP/GRP78 in its inactive form. On UPR activation, it becomes dissociated from the Bip/GRP78 complex and undergoes oligomerization and autophosphorylation, and thus becomes activated [124]. Active PERK plays an important role in suppressing global protein synthesis by attenuating mRNA translation and inhibiting the entry of new proteins into the ER lumen. This process is regulated by phosphorylation-mediated inactivation of the eukaryotic translation initiation factor 2 (eIF2α). Phosphorylation of eIF2α at Ser51 residue inhibits protein translation by reducing the Cyclin D1 pool and cell cycle arrest at the G1 phase, which ultimately diminishes protein burden and helps the cells to overcome the stressful conditions [125]. The activated PERK- eIF2α promotes the translation of ATF4, which encourages cell survival by regulating protein biosynthesis and transport. Cells lacking PERK are reported to be supersensitive to ER stress conditions. Thus, inhibition of the PERK- eIF2α-ATF4 signaling pathway could be a promising target for cancer prevention.

### 6.3. ATF6 Pathway

ATF6 is a type II transmembrane protein from the leucine zipper family of transcription factors [126]. It is the cytoprotective factor and ER stress modulator that participates actively in the UPR signaling pathway [127]. Under chronic ER stress, an isoform ATF6α disassociates from the GRP78 proteins and translocates to the Golgi apparatus, where it undergoes proteolysis by the resident Site 1 (S1P) and Site 2 (S2P) proteases. This releases a cytosolic fragment that migrates to the nucleus and regulates transcription [125]. To maintain ER hemostasis, cleaved ATF6α plays a prominent role in the regulation of genes involved in protein synthesis and ER-associated degradation (ERAD) [128].

## 7. Role of Kaempferol in ER Stress and Oxidative Stress-Induced Apoptosis

Uncontrolled and sustained ER stress leads to cellular damage and eventually induces apoptosis by activating the mitochondrial intrinsic apoptotic pathway. This pathway is activated by various micro-environmental stimuli, such as DNA damage, ER stress, ROS overload, and replication stress [129]. During prolonged ER stress, PERK induces selective translation of ATF4 and transcription of the CHOP gene, which ultimately results in the activation of apoptotic machinery [130]. Several studies have shown the importance of kaempferol in ER stress and oxidative stress-induced apoptosis through a different mechanism (Table 3). Kaempferol induces apoptosis in the liver cancer cell line via the ER stress-CHOP signaling pathway by increasing the protein expression levels of Grp78, Grp94, PERK, IRE1α, ATF6, caspase 4, CHOP, and cleaved caspase 3 [131]. Kaempferol pretreatment impedes hepatocyte apoptosis to protect mice from liver failure by regulating the ER stress-Grp78-CHOP signaling pathway [132]. Kaempferol has shown a protective effect in rat hepatoma cells over a broad concentration range by inducing oxidative stress and apoptosis [133]. Additionally, some researchers have revealed the antioxidant ability of kaempferol (present in aqueous Pepino leaf extract (AEPL)) on HepG2 cells by promoting the expression of Nrf2 and its target genes (*SOD1* and *GPX3*), reducing ER stress and inhibiting apoptosis [134].

## 8. Modulation of ER Stress and Autophagy Machinery by Kaempferol

Oxidative stress and ER stress tend to function through autophagy, which is a self-degradative process and plays an essential role in removing misfolded and degradative proteins and clearing damaged cellular organelles [135]. Interestingly, some researchers have found that autophagy itself is capable of preventing cancer in some phases via tissue damage and genomic instability, etc [136].

The ER, being a trafficking organelle, drives the cell towards death. The autophagy machinery is activated in response to ER stress [137]. Disruption in the autophagic process and alteration in ER hemostasis may promote serious liver diseases. Therefore, identifying and targeting the pathways with the help of traditional drugs appears to be beneficial in its treatment. Kaempferol exerts a positive effect on the autophagic machinery in combatting cancer [118]. Many researchers have demonstrated the modulatory effect of kaempferol on autophagy in different human cancers. In gastric cancer cells, kaempferol induces autophagic cell death via activating the IRE1-JNK-CHOP signaling pathway and inhibiting G9a cells [97]. Furthermore, another study validates the inhibitory effect of kaempferol in autophagy in lung cancer cells. Cells treated with kaempferol showed miR-340 overexpression, elevated PTEN, and reduced p-PI3K and p-AKT levels. The autophagic induction was confirmed through the increased expression of LC3-II, ATG7 and Beclin 1, and the reduced expression of p62 [18]. Kaempferol is found to inhibit cell proliferation, motility, and invasion by stimulating apoptosis and autophagy in RKO, HCT-116, HT-29, and DLD-1 colon cancer cell lines [138]. Kaempferol possesses anti-glioma activity by generating ROS and subsequently autophagy followed by pyroptosis (an inflammatory form of programmed cell death activated by some inflammasomes) in glioblastoma cell lines [83].

Kaempferol helps in modulating ER stress and autophagy, thus protecting the cells against malfunction [139]. Few reports are available indicating the potential of kaempferol in preventing HCC via autophagy and ER stress (Table 3). It induces cell mortality derived from autophagy by triggering the AMPK signaling pathway [140]. Kaempferol showed a concentration- and time-dependent inhibitory effect on liver cancer cells by inducing autophagy via the ER stress-CHOP signaling pathway [48].

## 9. Conclusions and Future Perspective

Kaempferol has been introduced into medical research due to its cancer-preventive activity. It specifically inhibits cancerous cells without disturbing the normal ones. It exerts chemopreventive effects against HCC by inducing mitochondrial apoptosis, autophagy, cell cycle arrest, ER stress, etc. The latest research on kaempferol shows it as an immune checkpoint modulator. Moreover, it can be used in combination with sorafenib and doxorubicin to enhance its efficacy in treating HCC and liver cancer.

Most of the research on the anti-cancer potential of kaempferol was carried out using human cell lines (in vitro). There are inadequate data on animal (in vivo) studies and clinical trials. There is an immense need for more in-depth in vivo experiments which will establish kaempferol as a more suitable and potent candidate as a chemopreventive agent against HCC.

Additionally, oxidative stress and ER stress both play a prominent role in different liver diseases, including HCC. Various reports show a close connection between oxidative stress and ER stress, but the molecular mechanism behind this association in hepatocarcinogenesis has not yet been completely explored. Therefore, further studies are required to determine the molecular mechanism of the interaction between OS and ER stress signaling in liver diseases.

Moreover, there are limitations in using kaempferol for the treatment of cancer, because of its poor solubility and bioavailability. This can be enhanced if it is given in combination with other anti-cancer drugs. Nanoformulations of kaempferol can also be prepared to increase its bioavailability.

## Figures and Tables

**Table 1:** Role of oxidative stress on HCC and associated diseases.

Cause	Factors Activated	Mechanism Involved	Impact on Oxidative Stress and HCC	References
**Hepatitis B Virus (HBV)**	HBx protein	↑ Oncogene expression, activation of macrophages to release proinflammatory cytokines (IL-1*β*, IL-6, CXCL-8, and TNF-*α*), activation of apoptosis	↑ ROS and HCC	[61]
	Gene mutation	Induce ER stress		
**Hepatitis C Virus (HCV)**	Core Protein	Activates signaling pathways (TNFR, PKR, and STAT3 pathways), induces apoptosis, metastasis, and DNA damage.	↑ ROS and HCC	[64,75]
	Fe2+ accumulation	Fenton reaction (Iron toxicity)		
**NASH**	Fatty toxicity	↑ IL-17	↑ ROS and HCC	[71]
	Central Obesity	Reduces the level of adiponectin, leading to increased cell growth, proliferation, and metastasis		

**Table 2:** The role of kaempferol in alleviating liver diseases.

Diseases Type	In Vitro/In Vivo Model	Mechanism of Action	Concentrations/Doses	References
**Alcoholic liver injury**	Mice	↑ expression of butyrate receptors, transporters, and TJ proteins in the intestinal mucosa.	25, 50 and 100 mg/kg	[83]
**Alcoholic liver injury**	ALI mice model	Increased antioxidant defense activity, decreased oxidative stress, and lipid peroxidation.	10 and 20 mg/kg	[82]
**Liver injury**	Bosentan-induced rat liver injury model and HEK-293 cells	Inhibition of OATP1B1 transporter, maintaining a level of AST, ALT	25 mg/kg and 1–150 μM	[84]
**Liver injury**	Male Swiss albino rats	Inhibition of lipid peroxidation caused by CCL4 reactive free radicals.	25 mg/kg	[85]
**Liver injury**	Male ddY mice	↓ TBARS and TNF-α level in CCL4 treated mice.	4.9 mg/kg	[80]
**Liver injury**	Mice and HepG2 cells	Reduces AA+Fe-induced ROS production and reversed glutathione depletion, ↓ cell death.	250 and 500 mg/kg and 100, 200 and 400 μM	[86]
**Liver fibrosis**	L02, LX2 and Rats	↓ Protein levels of cleaved caspase-3, ↑ p-ERK1/2, PI3K, and Bcl-XL protein expression in TNF-α-stimulated L02 cells. The suppressed proliferation of LX2 cells and up-regulation of Bax and cleaved caspase-8.	20 μM	[87]
**Liver fibrosis**	HSCs/Ccl4 induced mouse model	Down-regulation of hyaluronic acid, ALT, AST, Smad2/3. Inhibits collagen synthesis and activation of HSCs cells. Suppression of activin receptor-like kinase 5.	2–10 μmol/L	[88]
**Liver cancer**	HepG2	Apoptosis, reduced expression of miR-21, upregulation of PTEN expression and PI3K/AKT/mTOR signaling pathways inactivation.	0, 25, 50, 75, and 100 μM	[20]
**Liver cancer**	HepG2 cells	↑ PIG3 level at mRNA and protein level, ↑ROS production, cytochrome C release, ↓ mitochondrial membrane potential, upregulation of Bax/Bcl-2, activation of caspases-9 and -3, and maintaining the pro-oxidant activity.	10, 20, 40 and 80 μM	[89]
**Human hepatic cancer**	SK-HEP-1	↑ protein levels of p-AMPK, LC3-II, Atg 5, Atg 7, Atg 12 and Beclin 1, ↓ level of CDK1, cyclin B, p-AKT, and p-Mtor. Downregulation of CDK1/Cyclin B pathways, Induces autophagy.	0, 25, 50, 75 and 100 μM	[17]
**HCC**	Huh 7	HIF-1a activity inactivation by cytoplasmic mislocalization and MAPK pathway inhibition.	1–100 µM	[90]
**HCC**	HepG2	↑ The hypolipidemic effect through LDL-c uptake.	15 µM	[91]
**HCC**	HepG2 cells	↑ phosphorylation of JAK1, Tyk2, and STAT1/2, ↓ phosphorylation of STAT3, promoted endogenous IFN-α-regulated genes expression, ↓ expression of SOCS3, ↑the anti-proliferative effect of IFN-α, activation of the JAK/STAT signaling pathway	10 µg/mL	[92]
**Hepatotoxicity**	Male C57BL/6 mice	Decreased level of ALT, AST. Induce hepatocellular damage, ↑ expression of antioxidant enzymes, and apoptosis. Reduces NLRP3 expression and pro-inflammatory factors. Inhibition of HMGBI/TLR4/NF-KB signaling pathway.	30 and 60 mg/kg	[93]
**Acrylamide hepatic intoxication**	Wistar female albino rats	Reduced TBAR and GSH level	5, 10, 20 and 40 mg/kg	[94]
**Nonalcoholic steatohepatitis (NASH)**	Male C57BL/6 mice	↓ level of ALT, LDL, triglycerides, total cholesterol, lipid droplets and inflammatory cells infiltration in the liver, Upregulation of DEGs, Regulation of fatty acid degradation, expression of cytochrome P450, ↓ level of urinary proteins family (Mup17, Mup7, and Mup16).	4 mg/mL	[95]
**NAFLD**	HepG2 cells	↓ hepatic lipid accumulation, promote β oxidation in mitochondria and up-regulation of the expression of CPT1A	20 μg/mL	[96]

**Table 3:** Role of kaempferol in the induction of cell death (apoptosis/autophagy) through ER stress and oxidative stress pathways [48,49,50,51,52,53,54,55,56,57,58,59,60,61,62,63,64,65,66,67,68,69,70,71,72,73,74,75,76,77,78,79,80,81,82,83,84,85,86,87,88,89,90,91,92,93,94,95,96,97,98,99,100,101,102,103,104,105,106,107,108,109,110,111,112,113,114,115,116,117,118,119,120,121,122,123,124,125,126,127,128,129,130,131,132,133,134,135,136,137,138,139,140,141].

Diseases Type	In Vitro/In Vivo Model	Mechanism of Action	Concentations/Doses	References
**Acute liver failure**	Murine ALF model induced by D-galactosamine/lipopolysaccharide mice	Regulation of ER stress-Grp78-CHOP pathway	5 mg/kg	[132]
**HCC**	HepG2	Apoptosis, and Upregulation of CHOP gene expression.	0, 5, 10, 25 50 and 100 µM	[131]
**HCC**	H4IIE	H_2_O_2_ mediated lipid peroxidation leading to cell death and DNA damage, ↑ the activity of caspases-2, -3/7, -9, and -8/10, and apoptosis.	5–25 µM	[133]
**HCC**	HepG2 and Huh 7	↑ The protein level of Atg5, Atg7, Beclin1, and Overexpression of CHOP induces autophagy.	5~100 μM	[48]
**NASH**	HepG2 cells/C57BL/6 NASH mice model	Decresed expression of LXRα, LPCAT3 and ERS-related factors PERK, eIF2α, ATF6, ATF4, XBP1, CHOP, IRE1α and GRP78 and induction of apoptosis.	20, 40, 60 μmol/L and 20 mg/kg	[141]
**Hepatocellular lipotoxicity**	HepG2	Decreased ER stress, increased antioxidant ability and inhibited apoptosis.	1, 5, 10, 100 µg/mL	[134]
